# Supplementary material for: Universal protein misfolding intermediates can bypass the proteostasis network and remain soluble and less functional
Source: Nat Commun. 2022 Jun 2;13:3081. doi: 10.1038/s41467-022-30548-5 (PMC9163053; doi:10.1038/s41467-022-30548-5)
Supplement: Supplementary file 1 — Supplementary Information [file 41467_2022_30548_MOESM1_ESM.pdf]

Supplementary Information

Universal protein misfolding intermediates can bypass the proteostasis network and remain soluble and less functional

Daniel A. Nissley[1,ǂ], Yang Jiang[1,ǂ], Fabio Trovato[1], Ian Sitarik[1], Karthik B. Narayan[1], Philip To[2], Yingzi Xia[2], Stephen Fried[2,3], Edward P. O'Brien[1,4,5*]

[1]Department of Chemistry, Pennsylvania State University, University Park, PA 16802, USA
[2]Department of Chemistry, Johns Hopkins University, Baltimore, MD 21218, USA
[3]Thomas C. Jenkins Department of Biophysics, Johns Hopkins University, Baltimore, MD 21218, USA
[4]Bioinformatics and Genomics Graduate Program, The Huck Institutes of the Life Sciences, Pennsylvania State University, University Park, PA 16802, USA
[5]Institute for Computational and Data Sciences, Pennsylvania State University, University Park, PA 16802, USA
ǂ These authors contributed equally to this research project
* to whom correspondence should be addressed: epo2@psu.edu

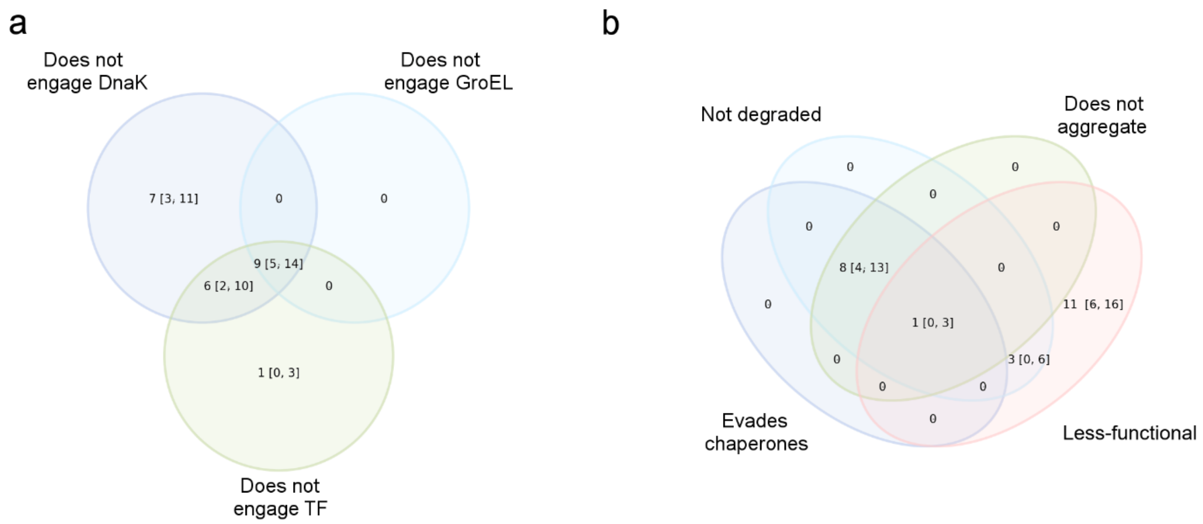

Supplementary Figure 1. Venn diagrams showing the overlap between Luciferase trajectories that are misfolded and (a) predicted to evade DnaK, GroEL, and TF or (b) predicted not to aggregate, not to be degraded, to evade all chaperones, and to remain non-functional. Error bars are 95% confidence intervals from bootstrapping 10^6 times.

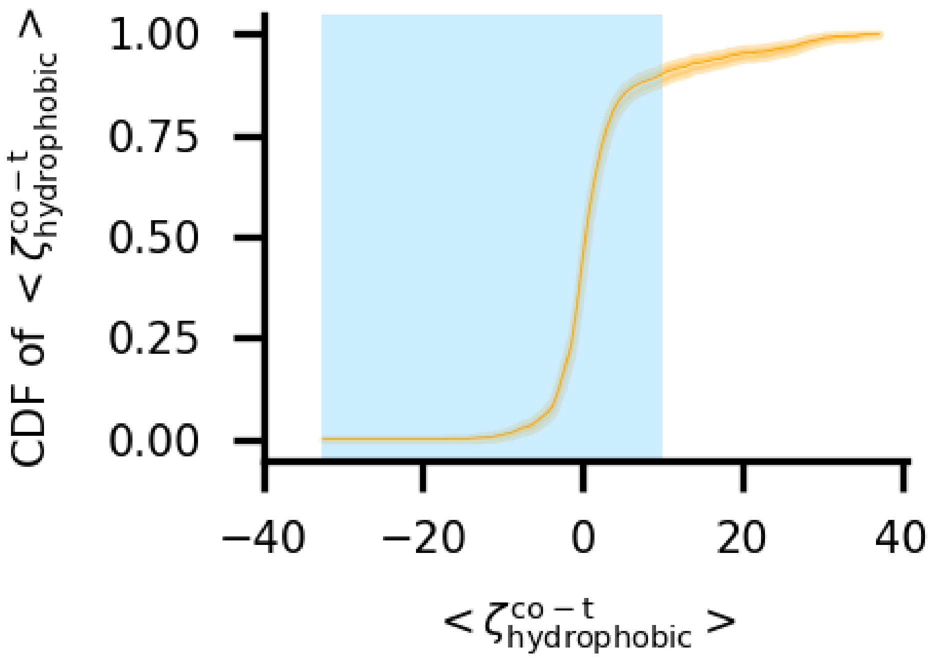

Supplementary Figure 2. Cumulative distribution function of $\langle\zeta^{\text{co-t}}_{\text{hydrophobic}}\rangle$ over the subset of 1,053 misfolded trajectories for which it was computed (see Methods). The blue shaded region indicates the subset of values taken to indicate no significant increase in trigger factor interactions relative to the folded population. The height of the CDF indicates the 95% confidence intervals.

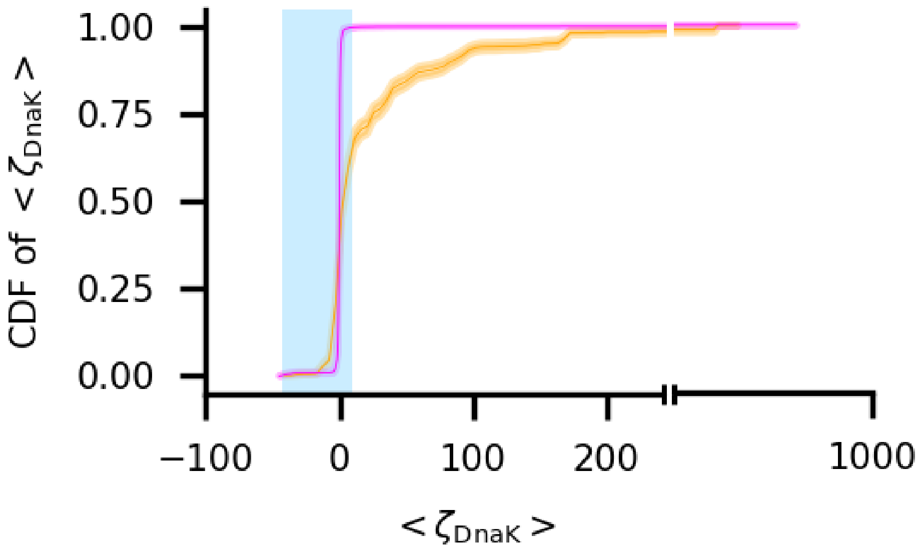

Supplementary Figure 3. Cumulative distribution function of $\langle \zeta_{\mathrm{DnaK}} \rangle$ over the subset of 1,631 misfolded (orange) and 4,469 folded (magenta) trajectories. The blue shaded region indicates the subset of values taken to indicate no significant increase in DnaK interactions relative to the native-state population. The height of the CDFs indicates the 95% confidence intervals.

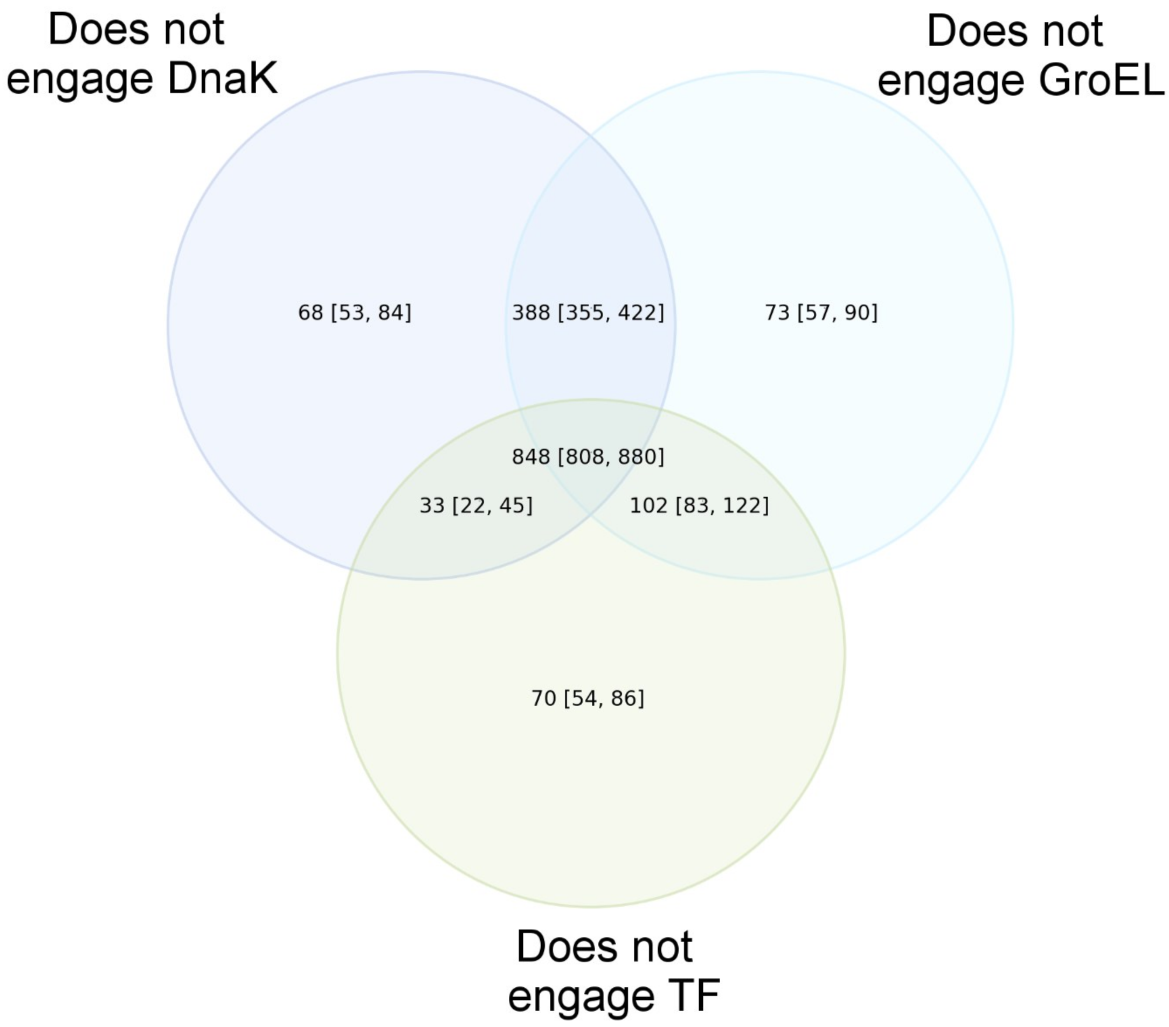

Supplementary Figure 4. Venn diagram showing the overlap between those trajectories that are predicted not to engage TF, DnaK, or GroEL/GroES. A total of 1,582 trajectories evade at least one chaperone. Error bars are 95% confidence intervals from bootstrapping 10^6 times.

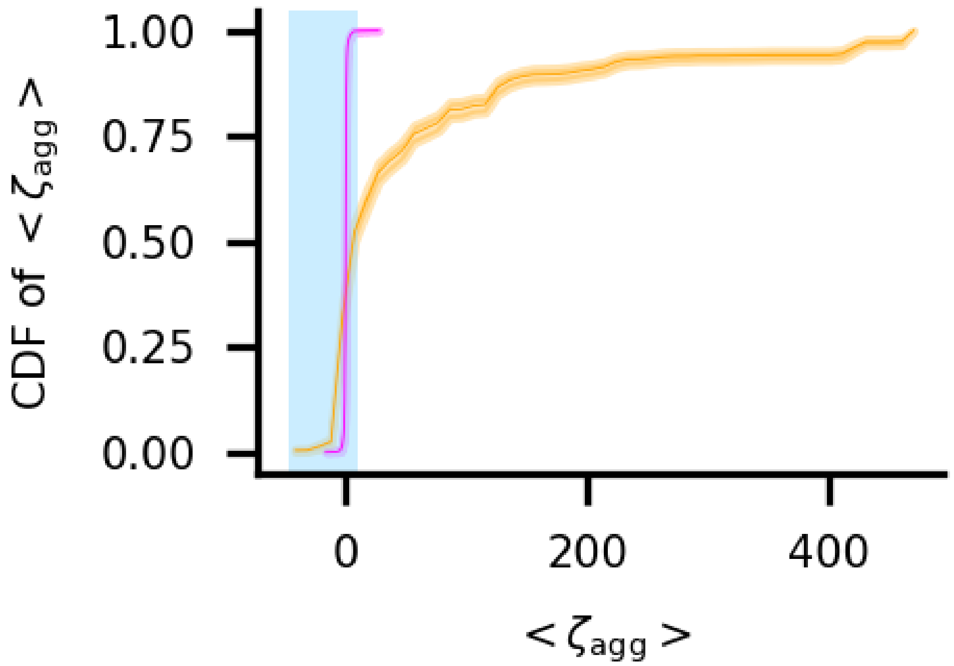

Supplementary Figure 5. Cumulative distribution function of $\langle \zeta_{\mathrm{Agg}} \rangle$ over the subset of 1,631 misfolded (orange) and 4,469 folded (magenta) trajectories. The blue shaded region indicates the subset of values taken to indicate no significant increase in aggregation propensity relative to the native state simulations. The height of the CDFs indicates the 95% confidence intervals.

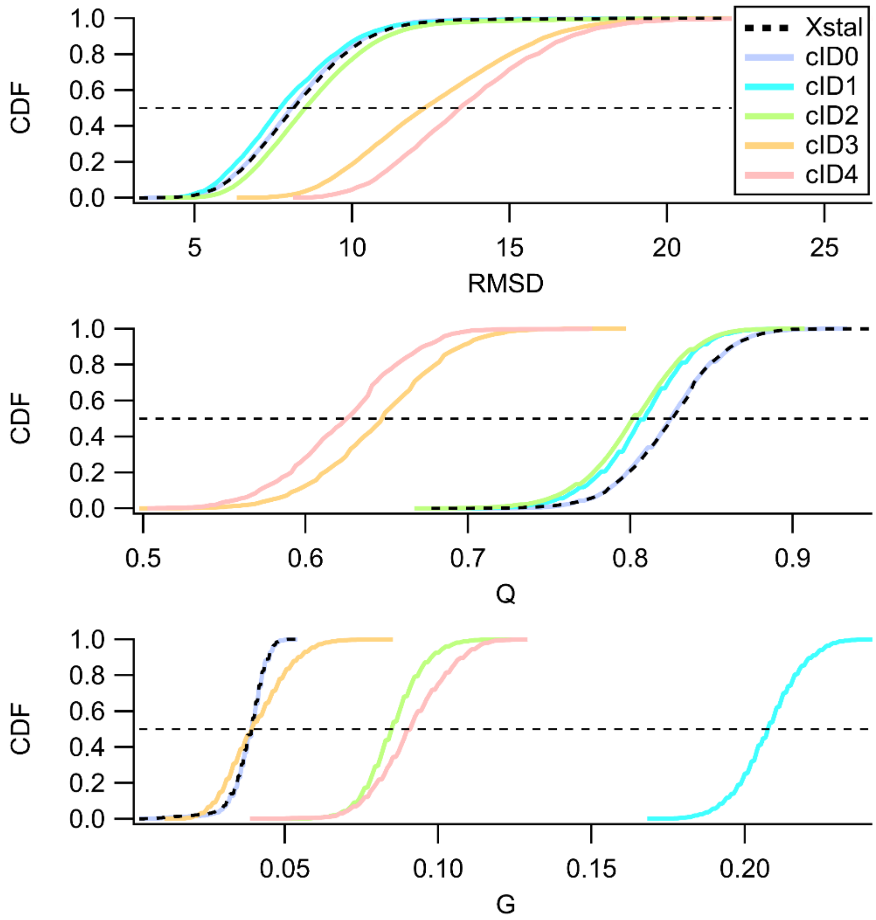

Supplementary Figure 6. The utility of the change in entanglement metric (G) for detecting structural perturbations not apparent by fraction of native contacts (Q) or root mean square deviation (RMSD) is exemplified by examining clusters of the trajectories for aminoimidazole ribonucleotide synthetase (PDB: 1CLI) relative to the cluster of trajectories started from the reference state. The 50 trajectories obtained from synthesis simulations can be clustered into 5 separate clusters characterized by the $\langle Q_{\text{mode}} \rangle$ and the corrected discrete distribution of a give type of change in entanglement (Eq. 7). cID0 contains 8/50 trajectories all of which terminate to the native state with no changes in entanglement. cID1 contains 3/50 trajectories that are near native like (i.e. $\langle Q_{\text{mode}} \rangle \approx \langle Q_{\text{mode}}^{NS} \rangle$) that show appreciable gain and loss of entanglements as well as pure switch in chirality. cID2 contains 32/50 trajectories that show appreciable gain and loss of entanglements with less near native like conformations (i.e. $\langle Q_{\text{mode}} \rangle \geq \langle Q_{\text{mode}}^{NS} \rangle - \sigma \langle Q_{\text{mode}}^{NS} \rangle$). cID3 contains 6/50 trajectories that show non-native like conformations (i.e. $\langle Q_{\text{mode}} \rangle < \langle Q_{\text{mode}}^{NS} \rangle - \sigma \langle Q_{\text{mode}}^{NS} \rangle$) with appreciable gain and loss of entanglements. cID4 contains 1/50 trajectories that show non-native like conformations with appreciable gain and loss of entanglements and pure change in chirality. For the clusters which contain near native like conformations and changes in entanglement of any kind (cID1 light blue and cID2) Q and RMSD fail to distinguish different clusters while G does not. For those clusters that contain non-native like conformations and changes in entanglement of any kind (cID3 & cID4) Q and RMSD are much more reliable.

Supplementary Table 1. Coarse-grain model parameters for PDB ID 4G36

PDB ID	Protein name	Domain or interface	Structural class	η used in model building
4G36	Firefly Luciferase	Domain 1:1-12; 356-437	β	1.442
		Domain 2:13-52; 212-355	α/β	1.114
		Domain 3: 53-211	α/β	1.114
		Domain 4: 438-550	α/β	1.916
		Interface 1|2	-	1.235
		Interface 1|3	-	1.235
		Interface 1|4	-	1.507*
		Interface 2|3	-	1.235
		Interface 2|4	-	1.235
		Interface 3|4	-	1.235

*indicates interface is unstable at all tested values of η and so median interface value from set of stable interfaces is used for all simulations

Supplementary Table 2 *E. coli* proteome dataset information

Index	PDB ID	Chain ID	UniProt Entry name	Protein name	Gene names
1	1A69	A	DEOD_ECOLI	Purine nucleoside phosphorylase DeoD-type	deoD, pup, b4384, JW4347
2	1A6J	B	PTSN_ECOLI	Nitrogen regulatory protein	ptsN, rpoP, yhbI, b3204, JW3171
3	1A82	A	BIOD1_ECOLI	ATP-dependent dethiobiotin synthetase BioD 1	bioD1, b0778, JW0761
4	1AG9	A	FLAV_ECOLI	Flavodoxin 1	fldA, b0684, JW0671
5	1AH9	Model 1	IF1_ECOLI	Translation initiation factor IF-1	infA, b0884, JW0867
6	1AKE	A	KAD_ECOLI	Adenylate kinase	Adk, dnaW, plsA, b0474, JW0463
7	1B9L	A	FOLX_ECOLI	Dihydroneopterin triphosphate 2'-epimerase	folX, b2303, JW2300
8	1CLI	A	PUR5_ECOLI	Phosphoribosylformylglycinamidine cyclo-ligase	purM, purG, b2499, JW2484
9	1D2F	A	MALY_ECOLI	Protein MalY	malY, b1622, JW1614
10	1DCJ	Model 1	TUSA_ECOLI	Sulfur carrier protein TusA	tusA, sirA, yhhP, b3470, JW3435
11	1DFU	P	RL25_ECOLI	50S ribosomal protein L25	rplY, b2185, JW2173
12	1DUV	G	OTC1_ECOLI	Ornithine carbamoyltransferase subunit I	argI, b4254, JW4211
13	1DXE	A	GARL_ECOLI	5-keto-4-deoxy-D-glucarate aldolase	garL, yhaF, b3126, JW3095
14	1EF9	A	SCPB_ECOLI	Methylmalonyl-CoA decarboxylase	scpB, mmcD, ygfG, b2919, JW2886
15	1EIX	C	PYRF_ECOLI	Orotidine 5'-phosphate decarboxylase	pyrF, b1281, JW1273
16	1EM8	A	HOLC_ECOLI	DNA polymerase III subunit chi	holC, b4259, JW4216
17	1EUM	A	FTNA_ECOLI	Bacterial non-heme ferritin	ftnA, ftn, gen-165, rsgA, b1905, JW1893
18	1FJJ	A	YBHB_ECOLI	UPF0098 protein YbhB	ybhB, b0773, JW0756
19	1FM0	D	MOAD_ECOLI	Molybdopterin synthase sulfur carrier subunit	moaD, chlA4, chlM, b0784, JW0767
20	1FTS	A	FTSY_ECOLI	Signal recognition particle receptor FtsY	ftsY, b3464, JW3429
21	1FUI	A	FUCI_ECOLI	L-fucose isomerase, FucIase	fucI, b2802, JW2773
22	1GER	B	GSHR_ECOLI	Glutathione reductase	gor, b3500, JW3467
23	1GLF	O	GLPK_ECOLI	Glycerol kinase	glpK, b3926, JW3897
24	1GQE	A	RF2_ECOLI	Peptide chain release factor RF2	prfB, supK, b2891, JW5847
25	1GQT	B	RBSK_ECOLI	Ribokinase	rbsK, b3752, JW3731
26	1GT7	A	RHAD_ECOLI	Rhamnulose-1-phosphate aldolase	rhaD, rhuA, b3902, JW3873
27	1GYT	L	AMPA_ECOLI	Cytosol aminopeptidase	pepA, carP, xerB, b4260, JW4217
28	1GZ0	C	RLMB_ECOLI	23S rRNA (guanosine-2'-O-)-methyltransferase RlmB	rlmB, yjfH, b4180, JW4138
29	1H16	A	PFLB_ECOLI	Formate acetyltransferase 1	pflB, pfl, b0903, JW0886
30	1H75	A	NRDH_ECOLI	Glutaredoxin-like protein NrdH	nrdH, ygaN, b2673, JW2648

Supplementary Table 2 cont.

Index	PDB ID	Chain ID	UniProt Entry name	Protein name	Gene names
31	1I6O	B	CAN_ECOLI	Carbonic anhydrase 2	can, cynT2, yadF, b0126, JW0122
32	1JNS	Model 1	PPIC_ECOLI	Peptidyl-prolyl cis-trans isomerase C	ppiC, parVA, b3775, JW3748
33	1JW2	A	HHA_ECOLI	Hemolysin expression-modulating protein Hha	hha, b0460, JW0449
34	1JX7	A	YCHN_ECOLI	Protein YchN	ychN, b1219, JW1210
35	1K7J	A	YCIO_ECOLI	Uncharacterized protein YciO	yciO, b1267, JW5196
36	1KO5	A	GNTK_ECOLI	Thermoresistant gluconokinase	gntK, b3437, JW3400
37	1KSF	X	CLPA_ECOLI	ATP-dependent Clp protease ATP-binding subunit ClpA	clpA, lopD, b0882, JW0866
38	1L6W	A	FSAA_ECOLI	Fructose-6-phosphate aldolase 1	fsaA, fsa, mipB, ybiZ, b0825, JW5109
39	1M3U	A	PANB_ECOLI	3-methyl-2-oxobutanoate hydroxymethyltransferase	panB, b0134, JW0130
40	1MZG	B	SUFE_ECOLI	Cysteine desulfuration protein SufE	sufE, ynhA, b1679, JW1669
41	1NAQ	A	CUTA_ECOLI	Divalent-cation tolerance protein CutA	cutA, cutA1, cycY, b4137, JW4097
42	1NG9	A	MUTS_ECOLI	DNA mismatch repair protein MutS	mutS, fdv, b2733, JW2703
43	1ORO	A	PYRE_ECOLI	Orotate phosphoribosyltransferase	pyrE, b3642, JW3617
44	1P7L	A	METK_ECOLI	S-adenosylmethionine synthase	metK, metX, b2942, JW2909
45	1P91	A	RLMA_ECOLI	23S rRNA (guanine(745)-N(1))-methyltransferase	rlmA, rrmA, yebH, b1822, JW1811
46	1PF5	A	YJGH_ECOLI	RutC family protein YjgH	yjgH, b4248, JW4206
47	1PMO	B	DCEB_ECOLI	Glutamate decarboxylase beta	gadB, b1493, JW1488
48	1PSU	B	PAAI_ECOLI	Acyl-coenzyme A thioesterase PaaI	paaI, ydbV, b1396, JW1391
49	1Q5X	A	RRAA_ECOLI	Regulator of ribonuclease activity A	rraA, menG, yiiV, b3929, JW3900
50	1QF6	A	SYT_ECOLI	Threonine-tRNA ligase	thrS, b1719, JW1709
51	1QTW	A	END4_ECOLI	Endonuclease 4	Nfo, b2159, JW2146
52	1RQJ	A	ISPA_ECOLI	Farnesyl diphosphate synthase	ispA, b0421, JW0411
53	1SG5	Model 1	ROF_ECOLI	Protein rof	Rof, yaeO, b0189, JW0184
54	1SV6	A	MHPD_ECOLI	2-keto-4-pentenoate hydratase	mhpD, b0350, JW0341
55	1SVT	J	CH60_ECOLI	60 kDa chaperonin	groL, groEL, mopA, b4143, JW4103
56	1T4B	A	DHAS_ECOLI	Aspartate-semialdehyde dehydrogenase	asd, hom, b3433, JW3396
57	1T8K	A	ACP_ECOLI	Acyl carrier protein	acpP, b1094, JW1080
58	1U0B	B	SYC_ECOLI	Cysteine--tRNA ligase	cysS, b0526, JW0515
59	1U60	A	GLSA1_ECOLI	Glutaminase 1	glsA1, ybaS, b0485, JW0474
60	1UUF	A	YAHK_ECOLI	Aldehyde reductase YahK	yahK, b0325, JW0317
61	1W78	A	FOLC_ECOLI	Dihydrofolate synthase/folylpolyglutamate synthase	folC, dedC, b2315, JW2312

Supplementary Table 2 cont.

Index	PDB ID	Chain ID	UniProt Entry name	Protein name	Gene names
62	1W8G	A	PLPHP_ECOLI	Pyridoxal phosphate homeostasis protein	yggS, b2951, JW2918
63	1WOC	C	PRIB_ECOLI	Primosomal replication protein N	priB, b4201, JW4159
64	1XN7	Model 1	FEOC_ECOLI	Probable [Fe-S]-dependent transcriptional repressor FeoC	feoC, yhgG, b3410, JW3373
65	1XRU	A	KDUI_ECOLI	4-deoxy-L-threo-5-hexosulose-uronate ketol-isomerase	kduI, yqeE, b2843, JW2811
66	1XVI	A	MPGP_ECOLI	Mannosyl-3-phosphoglycerate phosphatase	yedP, b1955, JW1938
67	1YQQ	A	XAPA_ECOLI	Purine nucleoside phosphorylase 2	xapA, pndA, b2407, JW2398
68	1ZYL	A	SRKA_ECOLI	Stress response kinase A	srkA, rdoA, yihE, b3859, JW3831
69	1ZZM	A	YJJV_ECOLI	Uncharacterized metal-dependent hydrolase YjjV	yjjV, b4378, JW4341
70	2A6Q	E	YOEB_ECOLI	Toxin YoeB	yoeB, b4539, JW5331
71	2AXD	Model 1	HOLE_ECOLI	DNA polymerase III subunit theta	holE, b1842, JW1831
72	2D1P	B	TUSC_ECOLI	Protein TusC	tusC, yheM, b3344, JW3306
73	2FEK	Model 1	WZB_ECOLI	Low molecular weight protein-tyrosine-phosphatase Wzb	wzb, b2061, JW2046
74	2FYM	A	ENO_ECOLI	Enolase	eno, b2779, JW2750
75	2GQR	A	PUR7_ECOLI	Phosphoribosylaminoimidazole-succinocarboxamide synthase	purC, b2476, JW2461
76	2H1F	A	A0A0H2VC26_ECOL6	Lipopolysaccharide heptosyltransferase-1	rfaC, c4447
77	2HD3	K	EUTN_ECOLI	Ethanolamine catabolic microcompartment shell protein EutN	eutN, cchB, yffY, b2456, JW2440
78	2HG2	A	ALDA_ECOLI	Lactaldehyde dehydrogenase	aldA, ald, b1415, JW1412
79	2HGK	Model 1	YQCC_ECOLI	Uncharacterized protein YqcC	yqcC, b2792, JW2763
80	2HNA	A	MIOC_ECOLI	Protein MioC	mioC, yieB, b3742, JW3720
81	2HNH	A	DPO3A_ECOLI	DNA polymerase III subunit alpha	dnaE, polC, b0184, JW0179
82	2HO9	Model 1	CHEW_ECOLI	Chemotaxis protein CheW	cheW, b1887, JW1876
83	2ID0	A	RNB_ECOLI	Exoribonuclease 2	rnb, b1286, JW1279
84	2JEE	C	ZAPB_ECOLI	Cell division protein ZapB	zapB, yiiU, b3928, JW3899
85	2JO6	Model 1	NIRD_ECOLI	Nitrite reductase (NADH) small subunit	nirD, b3366, JW3329
86	2JRX	Model 1	YEJL_ECOLI	UPF0352 protein YejL	yejL, b2187, JW2175
87	2KC5	Model 1	HYBE_ECOLI	Hydrogenase-2 operon protein HybE	hybE, b2992, JW2960
88	2KFW	Model 1	SLYD_ECOLI	FKBP-type peptidyl-prolyl cis-trans isomerase SlyD, PPIase	slyD, b3349, JW3311
89	2KX9	A	PT1_ECOLI	Phosphoenolpyruvate-protein phosphotransferase	ptsI, b2416, JW2409
90	2O1C	A	NUDB_ECOLI	Dihydroneopterin triphosphate diphosphatase	nudB, ntpA, b1865, JW1854
91	2OQ3	Model 1	PTMA_ECOLI	Mannitol-specific cryptic phosphotransferase enzyme IIA component	cmtB, b2934, JW2901
92	2PTH	A	PTH_ECOLI	Peptidyl-tRNA hydrolase	pth, b1204, JW1195
93	2PTQ	A	PUR8_ECOLI	Adenylosuccinate lyase	purB, b1131, JW1117
94	2QCU	B	GLPD_ECOLI	Aerobic glycerol-3-phosphate dehydrogenase	glpD, glyD, b3426, JW3389
95	2QVR	A	F16PA_ECOLI	Fructose-1,6-bisphosphatase class 1	fbp, fdp, b4232, JW4191

Supplementary Table 2 cont.

Index	PDB ID	Chain ID	UniProt Entry name	Protein name	Gene names
96	2R5N	A	TKT1_ECOLI	Transketolase 1	tktA, tkt, b2935, JW5478
97	2UYJ	A	TDCF_ECOLI	Putative reactive intermediate deaminase TdcF	tdcF, yhaR, b3113, JW5521
98	2V81	A	DGOA_ECOLI	2-dehydro-3-deoxy-6-phosphogalactonate aldolase	dgoA, yidU, b4477, JW5628
99	2WIU	A	HIPA_ECOLI	Ser/Thr-protein kinase HipA	hipA, b1507, JW1500
100	2WW4	A	ISPE_ECOLI	4-diphosphocytidyl-2-C-methyl-D-erythritol kinase, CMK	ispE, ipk, ychB, b1208, JW1199
101	2YVA	A	DIAA_ECOLI	DnaA initiator-associating protein DiaA	diaA, yraO, b3149, JW3118
102	3ASV	B	YDFG_ECOLI	NADP-dependent 3-hydroxy acid dehydrogenase YdfG	ydfG, b1539, JW1532
103	3BMB	B	RNK_ECOLI	Regulator of nucleoside diphosphate kinase	rnk, b0610, JW0602
104	3BRQ	B	ASCG_ECOLI	HTH-type transcriptional regulator AscG	ascG, b2714, JW5434
105	3GN5	B	MQSA_ECOLI	Antitoxin MqsA	mqsA, ygiT, b3021, JW2989
106	3HWO	A	ENTC_ECOLI	Isochorismate synthase EntC	entC, b0593, JW0585
107	3IV5	B	FIS_ECOLI	DNA-binding protein Fis	fis, b3261, JW3229
108	3M7M	X	HSLO_ECOLI	33 kDa chaperonin	hslO, yrfI, b3401, JW5692
109	3N1S	J	HINT_ECOLI	Purine nucleoside phosphoramidase	hinT, ycfF, b1103, JW1089
110	3NXC	A	SLMA_ECOLI	Nucleoid occlusion factor SlmA	slmA, ttk, yicB, b3641, JW5641
111	3OFO	D	RS4_ECOLI	30S ribosomal protein S4	rpsD, ramA, b3296, JW3258
112	3PCO	D	SYFB_ECOLI	Phenylalanine--tRNA ligase beta subunit	pheT, b1713, JW1703
113	3QOU	A	CNOX_ECOLI	Chaperedoxin	cnoX, ybbN, b0492, JW5067
114	4A2C	A	GATD_ECOLI	Galactitol 1-phosphate 5-dehydrogenase	gatD, b2091, JW2075
115	4DCM	A	RLMG_ECOLI	Ribosomal RNA large subunit methyltransferase G	rlmG, ygjO, b3084, JW5513
116	4DZD	A	CAS6_ECOLI	CRISPR system Cascade subunit CasE	casE, cas6e, ygcH, b2756, JW2726
117	4E8B	A	RSME_ECOLI	Ribosomal RNA small subunit methyltransferase E	rsmE, yggJ, b2946, JW2913
118	4FZW	A	PAAF_ECOLI	2,3-dehydroadipyl-CoA hydratase	paaF, ydbR, b1393, JW1388
119	4HR7	A	ACCC_ECOLI	Biotin carboxylase	accC, fabG, b3256, JW3224
120	4IM7	A	A0A0H2V7F2_ECOL6	Hypothetical oxidoreductase ydfI	c1968
121	4IWX	A	RIMK_ECOLI	Ribosomal protein S6--L-glutamate ligase	rimK, b0852, JW0836
122	4KN7	A	RPOB_ECOLI	DNA-directed RNA polymerase subunit beta, RNAP subunit beta	rpoB, groN, nitB, rif, ron, stl

Supplementary Table 3. Proteins categorized by percent of trajectories misfolded

Category	Number of proteins	PDB IDs
At least 2% misfolded	80	1A69, 1A6J, 1A82, 1AH9, 1DFU, 1DXE, 1FJJ, 1GQT, 1GT7, 1H16, 1I6O, 1K7J, 1L6W, 1M3U, 1NAQ, 1ORO, 1P91, 1PF5, 1PMO, 1Q5X, 1SV6, 1U60, 1W8G, 1WOC, 1YQQ, 1ZYL, 2HNA, 2JO6, 2O1C, 2PTH, 2V81, 2YVA, 3BMB, 3HWO, 4A2C, 1CLI, 1D2F, 1DUV, 1EF9, 1FTS, 1FUI, 1GER, 1GLF, 1GYT, 1GZ0, 1KSF, 1NG9, 1P7L, 1QF6, 1SVT, 1T4B, 1U0B, 1UUF, 1W78, 1XRU, 1XVI, 2FYM, 2H1F, 2HG2, 2HNH, 2ID0, 2KFW, 2KX9, 2PTQ, 2QCU, 2R5N, 2WIU, 2WW4, 3BRQ, 3GN5, 3M7M, 3PCO, 4DCM, 4DZD, 4E8B, 4FZW, 4HR7, 4IM7, 4IWX, 4KN7
At least 20% misfolded	49	1A69, 1DFU, 1DXE, 1GQT, 1H16, 1K7J, 1P91, 1SV6, 1U60, 1YQQ, 1ZYL, 2HNA, 2JO6, 2V81, 3HWO, 4A2C, 1CLI, 1DUV, 1FTS, 1FUI, 1GLF, 1GYT, 1GZ0, 1KSF, 1NG9, 1P7L, 1QF6, 1SVT, 1T4B, 1U0B, 1W78, 1XRU, 1XVI, 2FYM, 2HG2, 2HNH, 2ID0, 2KFW, 2KX9, 2QCU, 2R5N, 2WIU, 3GN5, 3PCO, 4E8B, 4HR7, 4IM7, 4IWX, 4KN7
At least 50% misfolded	27	1GQT, 1H16, 1K7J, 1P91, 1U60, 1YQQ, 2JO6, 2V81, 1CLI, 1FUI, 1GYT, 1GZ0, 1KSF, 1P7L, 1T4B, 1U0B, 1XVI, 2HNH, 2ID0, 2KX9, 2QCU, 2R5N, 3PCO, 4E8B, 4HR7, 4IM7, 4KN7
100% misfolded	9	1GQT, 1H16, 1FUI, 1GYT, 1GZ0, 1XVI, 4E8B, 4HR7, 4KN7

Supplementary Table 4. Extrapolated folding times for the 73 proteins with a reliable estimate

PDB ID	Simulated τ_F, ns	Estimated experimental τ_F, s	Estimated experimental τ_F, min	Estimated experimental τ_F, h	Estimated experimental τ_F, d
1SG5	1.08E+01	4.29E-02	7.15E-04	1.19E-05	4.97E-07
2AXD	1.08E+01	4.29E-02	7.15E-04	1.19E-05	4.97E-07
3IV5	1.15E+01	4.55E-02	7.58E-04	1.26E-05	5.27E-07
2JRX	1.57E+01	6.22E-02	1.04E-03	1.73E-05	7.20E-07
2KC5	1.68E+01	6.67E-02	1.11E-03	1.85E-05	7.72E-07
2HO9	2.10E+01	8.34E-02	1.39E-03	2.32E-05	9.65E-07
2JEE	2.28E+01	9.05E-02	1.51E-03	2.52E-05	1.05E-06
3QOU	3.77E+01	1.50E-01	2.49E-03	4.16E-05	1.73E-06
1H75	4.25E+01	1.69E-01	2.81E-03	4.68E-05	1.95E-06
1T8K	4.30E+01	1.70E-01	2.84E-03	4.73E-05	1.97E-06
2FEK	4.30E+01	1.70E-01	2.84E-03	4.74E-05	1.97E-06
1MZG	5.86E+01	2.32E-01	3.87E-03	6.45E-05	2.69E-06
3NXC	9.97E+01	3.96E-01	6.60E-03	1.10E-04	4.58E-06
1FM0	1.07E+02	4.26E-01	7.10E-03	1.18E-04	4.93E-06
2HGK	2.84E+02	1.13E+00	1.88E-02	3.13E-04	1.30E-05
4DCM	2.92E+02	1.16E+00	1.93E-02	3.21E-04	1.34E-05
1GQE	3.42E+02	1.36E+00	2.26E-02	3.76E-04	1.57E-05
1ZZM	3.51E+02	1.39E+00	2.32E-02	3.87E-04	1.61E-05
2KX9	3.55E+02	1.41E+00	2.35E-02	3.92E-04	1.63E-05
2OQ3	3.63E+02	1.44E+00	2.40E-02	4.00E-04	1.67E-05
1EIX	4.34E+02	1.72E+00	2.87E-02	4.78E-04	1.99E-05
1KO5	4.39E+02	1.74E+00	2.90E-02	4.84E-04	2.02E-05
1GT7	5.36E+02	2.13E+00	3.54E-02	5.91E-04	2.46E-05
3N1S	5.83E+02	2.31E+00	3.85E-02	6.42E-04	2.68E-05
2QVR	6.26E+02	2.48E+00	4.14E-02	6.90E-04	2.87E-05
1AG9	8.60E+02	3.41E+00	5.68E-02	9.47E-04	3.95E-05
1EUM	9.16E+02	3.64E+00	6.06E-02	1.01E-03	4.21E-05
3OFO	9.94E+02	3.94E+00	6.57E-02	1.10E-03	4.56E-05
2KFW	1.81E+03	7.20E+00	1.20E-01	2.00E-03	8.33E-05
3BMB	3.36E+03	1.33E+01	2.22E-01	3.70E-03	1.54E-04
2PTH	3.39E+03	1.35E+01	2.24E-01	3.74E-03	1.56E-04
2GQR	3.82E+03	1.51E+01	2.52E-01	4.21E-03	1.75E-04
1QTW	4.13E+03	1.64E+01	2.73E-01	4.55E-03	1.90E-04
1JX7	4.43E+03	1.76E+01	2.93E-01	4.88E-03	2.03E-04
2YVA	5.42E+03	2.15E+01	3.59E-01	5.98E-03	2.49E-04
1PSU	1.05E+04	4.16E+01	6.93E-01	1.15E-02	4.81E-04
1GLF	1.28E+04	5.09E+01	8.49E-01	1.41E-02	5.89E-04
1A6J	1.54E+04	6.12E+01	1.02E+00	1.70E-02	7.09E-04
1DXE	1.81E+04	7.18E+01	1.20E+00	2.00E-02	8.31E-04
2HD3	1.93E+04	7.65E+01	1.28E+00	2.13E-02	8.86E-04
4IM7	2.46E+04	9.77E+01	1.63E+00	2.71E-02	1.13E-03
1XRU	3.38E+04	1.34E+02	2.23E+00	3.72E-02	1.55E-03

Supplementary Table 4 cont.

PDB ID	Simulated τ_F, ns	Estimated experimental τ_F, s	Estimated experimental τ_F, min	Estimated experimental τ_F, h	Estimated experimental τ_F, d
1I6O	4.03E+04	1.60E+02	2.66E+00	4.44E-02	1.85E-03
1EF9	4.66E+04	1.85E+02	3.08E+00	5.13E-02	2.14E-03
1ORO	4.82E+04	1.91E+02	3.18E+00	5.31E-02	2.21E-03
1SV6	4.94E+04	1.96E+02	3.26E+00	5.44E-02	2.27E-03
1AH9	6.57E+04	2.61E+02	4.34E+00	7.24E-02	3.02E-03
3GN5	7.30E+04	2.90E+02	4.83E+00	8.04E-02	3.35E-03
1ZYL	8.02E+04	3.18E+02	5.30E+00	8.84E-02	3.68E-03
2PTQ	5.28E+05	2.09E+03	3.49E+01	5.82E-01	2.42E-02
2V81	5.58E+05	2.22E+03	3.69E+01	6.15E-01	2.56E-02
1FTS	4.60E+06	1.83E+04	3.04E+02	5.07E+00	2.11E-01
3M7M	7.33E+13	2.91E+11	4.85E+09	8.08E+07	3.36E+06
4IWX	7.61E+13	3.02E+11	5.03E+09	8.39E+07	3.50E+06
1KSF	2.59E+14	1.03E+12	1.71E+10	2.85E+08	1.19E+07
3BRQ	3.83E+18	1.52E+16	2.53E+14	4.22E+12	1.76E+11
2FYM	1.28E+19	5.06E+16	8.44E+14	1.41E+13	5.86E+11
1CLI	2.35E+19	9.31E+16	1.55E+15	2.59E+13	1.08E+12
2O1C	3.30E+19	1.31E+17	2.18E+15	3.64E+13	1.52E+12
1NG9	1.35E+20	5.37E+17	8.94E+15	1.49E+14	6.21E+12
1U60	1.65E+20	6.53E+17	1.09E+16	1.81E+14	7.56E+12
1K7J	6.72E+20	2.67E+18	4.45E+16	7.41E+14	3.09E+13
1W78	7.66E+20	3.04E+18	5.07E+16	8.45E+14	3.52E+13
2HNH	7.85E+20	3.11E+18	5.19E+16	8.65E+14	3.60E+13
1U0B	2.82E+21	1.12E+19	1.87E+17	3.11E+15	1.30E+14
1UUF	7.80E+21	3.09E+19	5.15E+17	8.59E+15	3.58E+14
2ID0	4.21E+22	1.67E+20	2.78E+18	4.64E+16	1.93E+15
1QF6	7.43E+22	2.95E+20	4.91E+18	8.19E+16	3.41E+15
2HG2	2.83E+23	1.12E+21	1.87E+19	3.12E+17	1.30E+16
2QCU	1.66E+24	6.60E+21	1.10E+20	1.83E+18	7.64E+16
2WIU	4.80E+27	1.91E+25	3.18E+23	5.29E+21	2.20E+20
1GER	3.73E+28	1.48E+26	2.47E+24	4.11E+22	1.71E+21
1YQQ	2.34E+29	9.30E+26	1.55E+25	2.58E+23	1.08E+22

Supplementary Table 5. Proteins with at least one misfolded trajectory expected to bypass chaperones

Category	Number of unique proteins	PDB IDs
Not expected to interact with TF	70	2ID0, 4IWX, 1A69, 2WIU, 1U60, 4DZD, 2HNH, 3HWO, 1KSF, 1ORO, 1ZYL, 3BRQ, 1XRU, 1EF9, 2PTQ, 2FYM, 1NG9, 1DXE, 2JO6, 4A2C, 1PMO, 2KX9, 2R5N, 1K7J, 1GT7, 2WW4, 3M7M, 3PCO, 1GLF, 1GER, 2H1F, 1YQQ, 1DFU, 1SVT, 2V81, 1NAQ, 1U0B, 1PF5, 1FJJ, 2YVA, 1DUV, 1UUF, 3BMB, 1A6J, 1SV6, 1W78, 4IM7, 2KFW, 1Q5X, 1P7L, 1QF6, 1T4B, 4FZW, 1AH9, 1FTS, 2QCU, 1CLI, 2PTH, 2O1C, 2HNA, 1W8G, 3GN5, 1WOC, 4DCM, 1A82, 2HG2, 1L6W, 1D2F, 1I6O, 1M3U
Not expected to interact with GroEL/GroES	80	2ID0, 1XVI, 4IWX, 1A69, 1FUI, 2WIU, 1U60, 4DZD, 2HNH, 3HWO, 1KSF, 1ORO, 1P91, 1ZYL, 3BRQ, 1XRU, 1EF9, 2PTQ, 2FYM, 1NG9, 1DXE, 4HR7, 2JO6, 2HG2, 4A2C, 1PMO, 2KX9, 2R5N, 1K7J, 1GT7, 1GQT, 2WW4, 3M7M, 3PCO, 1GLF, 1GER, 2H1F, 1YQQ, 1DFU, 1SVT, 2V81, 1NAQ, 1U0B, 1PF5, 1FJJ, 2YVA, 1DUV, 1UUF, 3BMB, 1A6J, 1H16, 1SV6, 1W78, 4IM7, 2KFW, 1Q5X, 1P7L, 1QF6, 2PTH, 3GN5, 4FZW, 1AH9, 1FTS, 2QCU, 1GZ0, 1CLI, 2O1C, 2HNA, 4E8B, 1W8G, 1WOC, 4DCM, 1A82, 1L6W, 1T4B, 1D2F, 1I6O, 4KN7, 1GYT, 1M3U
Not expected to interact with DnaK	77	2ID0, 1XVI, 4IWX, 1A69, 1FUI, 2WIU, 1U60, 4DZD, 2HNH, 3HWO, 1KSF, 1ORO, 1P91, 1ZYL, 3BRQ, 1XRU, 2PTQ, 2FYM, 4HR7, 1NG9, 1DXE, 2JO6, 2HG2, 4A2C, 1PMO, 2KX9, 2R5N, 1K7J, 1GT7, 2WW4, 3M7M, 3PCO, 1GLF, 1GER, 2H1F, 1YQQ, 1DFU, 1SVT, 2V81, 1NAQ, 1U0B, 1PF5, 1FJJ, 2YVA, 1DUV, 1UUF, 3BMB, 1A6J, 1H16, 1SV6, 1W78, 4IM7, 2KFW, 1Q5X, 1P7L, 1QF6, 2PTH, 3GN5, 4FZW, 1AH9, 1FTS, 2QCU, 1GZ0, 2O1C, 2HNA, 1GYT, 4E8B, 1W8G, 1WOC, 4DCM, 1A82, 1L6W, 1T4B, 1D2F, 4KN7, 1CLI, 1M3U
Not expected to interact with TF, GroEL/GroES, or DnaK	68	2ID0, 4IWX, 1A69, 2WIU, 1U60, 4DZD, 2HNH, 3HWO, 1KSF, 1ORO, 1ZYL, 3BRQ, 1XRU, 2PTQ, 2FYM, 1NG9, 1DXE, 2JO6, 4A2C, 1PMO, 2KX9, 2R5N, 1K7J, 1GT7, 2WW4, 3M7M, 3PCO, 1GLF, 1GER, 2H1F, 1YQQ, 1DFU, 1SVT, 2V81, 1NAQ, 1U0B, 1PF5, 1FJJ, 2YVA, 1DUV, 1UUF, 3BMB , 1A6J, 1SV6, 1W78, 4IM7, 2KFW, 1Q5X, 1P7L, 1QF6, 4FZW, 1AH9, 1FTS, 2QCU, 2PTH, 2O1C, 2HNA, 1W8G, 3GN5, 1WOC, 4DCM, 1A82, 2HG2, 1L6W, 1T4B, 1D2F, 1CLI, 1M3U

Supplementary Table 6. Proteins with at least one misfolded trajectory expected not to aggregate or be degraded

Category	Number of unique proteins	PDB IDs
Not expected to aggregate	68	2ID0, 1XVI, 1A69, 1FUI, 2WIU, 1U60, 4DZD, 2HNH, 3HWO, 1KSF, 1ORO, 1P91, 1ZYL, 3BRQ, 1XRU, 2PTQ, 2FYM, 4HR7, 1NG9, 2JO6, 2HG2, 4A2C, 1PMO, 2KX9, 2R5N, 1K7J, 1GT7, 2WW4, 3M7M, 3PCO, 1DFU, 1SVT, 1U0B, 1PF5, 1FJJ, 1DUV, 1SV6, 1W78, 4IM7, 1YQQ, 2KFW, 1Q5X, 1P7L, 1QF6, 2PTH, 3GN5, 4FZW, 1AH9, 1FTS, 2QCU, 1GZ0, 1CLI, 2O1C, 2HNA, 1A6J, 1W8G, 1WOC, 4DCM, 1A82, 1L6W, 1T4B, 1D2F, 1NAQ, 1I6O, 1GER, 4KN7, 1GYT, 1M3U
Not expected to be degraded	70	2ID0, 1XVI, 1A69, 1FUI, 2WIU, 1U60, 4DZD, 2HNH, 3HWO, 1KSF, 1ORO, 1P91, 1ZYL, 3BRQ, 1XRU, 2PTQ, 2FYM, 1NG9, 4HR7, 2JO6, 2HG2, 4A2C, 1PMO, 2KX9, 2R5N, 1K7J, 1GT7, 2WW4, 3M7M, 3PCO, 1GER, 1YQQ, 1DFU, 1SVT, 1U0B, 1PF5, 1FJJ, 1DUV, 1SV6, 1W78, 4IM7, 2KFW, 1Q5X, 1NAQ, 1P7L, 1QF6, 2PTH, 3GN5, 4FZW, 1AH9, 1FTS, 2QCU, 1GZ0, 1CLI, 2O1C, 1A6J, 4E8B, 1W8G, 1WOC, 4DCM, 1A82, 2HNA, 1L6W, 1T4B, 1UUF, 1D2F, 1I6O, 4KN7, 1GYT, 1M3U

Supplementary Table 7. Proteins with misfolded conformations expected to bypass proteostasis machinery and remain soluble but less functional

Category	Number of unique proteins	PDB IDs
Expected to bypass chaperones, not aggregate, and not be degraded	58	2ID0, 1A69, 2WIU, 1U60, 4DZD, 2HNH, 3HWO, 1KSF, 1ORO, 1ZYL, 3BRQ,1XRU, 2PTQ, 2FYM, 1NG9, 2JO6, 4A2C, 1PMO, 2KX9, 2R5N, 1K7J, 1GT7, 2WW4, 3M7M, 3PCO, 1DFU, 1SVT, 1U0B, 1PF5, 1FJJ, 1DUV, 1SV6, 1W78, 4IM7, 1YQQ, 2KFW, 1Q5X, 1P7L, 1QF6, 4FZW, 1AH9, 1FTS, 2QCU, 2PTH, 2O1C, 1A6J, 1W8G, 3GN5, 1WOC, 4DCM, 1A82, 2HNA, 1L6W, 1T4B, 1D2F, 1GER, 1CLI, 1M3U
Expected to exhibit reduced function	69	1XVI, 4IWX, 1A69, 1FUI, 2WIU, 1U60, 4DZD, 2HNH, 3HWO, 2ID0, 1ZYL, 1XRU, 1P91, 1EF9, 2FYM, 4HR7, 1NG9, 1DXE, 2HG2, 1PMO, 2R5N, 1K7J, 1GQT, 3PCO, 1GLF, 2H1F, 1YQQ, 1DFU, 1SVT, 2V81, 1NAQ, 1U0B, 2YVA, 4KN7, 1DUV, 1UUF, 3BMB, 1A6J, 1H16, 1SV6, 1W78, 4IM7, 1Q5X, 2PTH, 1T4B, 4FZW, 1FTS, 2QCU, 1P7L, 1GZ0, 1KSF, 1CLI, 1GYT, 1WOC, 2PTQ, 1A82, 2WW4, 4A2C, 3GN5, 1L6W, 1GT7, 1QF6, 1D2F, 1I6O, 1GER, 2KX9, 2O1C, 1PF5, 3M7M
Expected to bypass proteostasis machinery and to exhibit reduced function	41	1A69, 2WIU, 1U60, 4DZD, 3HWO, 1ZYL, 1XRU, 2FYM, 1NG9, 1PMO, 2R5N, 1K7J, 1DFU, 1SVT, 1U0B, 1SV6, 1W78, 4IM7, 1YQQ, 1Q5X, 4FZW, 1FTS, 2QCU, 1KSF, 2PTH, 2HNH, 1WOC, 2PTQ, 1A82, 2ID0, 3GN5, 3PCO, 1L6W, 1T4B, 1A6J, 1GT7, 1D2F, 1GER, 1DUV, 2O1C, 1PF5

Supplementary Table 8. Values of $\langle \zeta_{\text{peptide}} \rangle$ and locations of principle entanglements by metastable state

Markov State	$\langle \zeta_{\text{peptide}} \rangle$, %			Principle entanglement*		
	**[333-354]**	**[F351]**	**[L293]**	**Loop segment**	**Threading segment**	**Overlapping with non-refoldable region?**
S1	1.56 [0.83, 2.30]	108.38 [84.49, 133.75]	50.45 [-12.64, 123.61]	**44-314**	**318-337**	Yes
S2	86.53 [84.74, 88.30]	6446.30 [6266.09, 6626.00]	28.83 [-27.88, 99.59]	271-288	218-237	No
S3	1.42 [1.11, 1.74]	51.22 [42.10, 60.67]	-12.30 [-42.09, 22.20]	-	-	-
S4	14.66 [13.73, 15.60]	1802.97 [1707.65, 1900.01]	358.21 [151.55, 608.55]	90-138	**318-337**	Yes
S5	22.42 [21.41, 23.43]	1644.79 [1567.43, 1723.38]	58.64 [-8.93, 154.10]	271-288	218-237	No
S6	1.03 [0.38, 1.69]	358.65 [312.01, 407.32]	87.72 [-2.56, 212.08]	41-207	**318-337**	Yes
S7	6.55 [6.41, 6.69]	506.27 [496.06, 516.54]	155.52 [125.11, 188.00]	**44-313**	**317-336**	Yes
S8	3.85 [3.61, 4.10]	302.66 [290.37, 315.22]	66.70 [25.23, 115.01]	**44-313**	**317-336**	Yes

*principle entanglement refers to the entanglement present in a representative structure for each metastable state. Segments that overlap with [333-354], [F351], or [L293] are highlighted in bold.

Supplementary Table 9. Definitions of entanglement types

Type	Change in entanglement	Change in chirality	Conditional*
G0	Gain	No	$\lvert g^{\text{current}}(i,j)\rvert > \lvert g^{\text{native}}(i,j)\rvert$ and $g^{\text{current}}(i,j) \times g^{\text{native}}(i,j) \geq 0$
G1	Gain	Yes	$\lvert g^{\text{current}}(i,j)\rvert > \lvert g^{\text{native}}(i,j)\rvert$ and $g^{\text{current}}(i,j) \times g^{\text{native}}(i,j) < 0$
G2	Lose	No	$\lvert g^{\text{current}}(i,j)\rvert < \lvert g^{\text{native}}(i,j)\rvert$ and $g^{\text{current}}(i,j) \times g^{\text{native}}(i,j) \geq 0$
G3	Lose	Yes	$\lvert g^{\text{current}}(i,j)\rvert < \lvert g^{\text{native}}(i,j)\rvert$ and $g^{\text{current}}(i,j) \times g^{\text{native}}(i,j) < 0$
G4	None	Yes	$\lvert g^{\text{current}}(i,j)\rvert = \lvert g^{\text{native}}(i,j)\rvert$ and $g^{\text{current}}(i,j) \times g^{\text{native}}(i,j) < 0$
G5	None	None	$\lvert g^{\text{current}}(i,j)\rvert = \lvert g^{\text{native}}(i,j)\rvert$ and $g^{\text{current}}(i,j) \times g^{\text{native}}(i,j) \geq 0$

* conditional used to determine if a native contact between residues i and j has a change in a given type of entanglement in the current structure relative to the native state structure.

Supplementary Table 10. Functional residues database terms and descriptions

Term in database	Description
PDB used in model	Uniprot: PDB ID used in the simulation
Gene name	Uniprot: Name of gene
Protein Uniprot ID	Uniprot: ID associated with gene in uniprot
Ordered Locus	Uniprot: Ordered locus associated with gene
Function	Uniprot: Function of the gene product
Catalytic activity	Uniprot: Catalytic activity of protein
Subunit	Uniprot: Information about n-mers structure
GO functions	Uniprot: Gene ontology functions
Binding site	Uniprot: Information about mapped and unmapped binding site
Active site	Uniprot: Information about mapped and unmapped active site
Metal binding site	Uniprot: Information about mapped and unmapped metal binding site
Site	Uniprot: Information about mapped and unmapped site
Absorption	Uniprot: Information about ligands absorbed
Calcium binding	Uniprot: Information about calcium binding
DNA binding	Uniprot: Information about DNA binding
EC number	Uniprot: Enzyme Commission number
Metal binding	Uniprot: Information about mapped and unmapped metal binding
Nucleotide binding	Uniprot: Information about mapped and unmapped about nucleotide binding
Pathway	Uniprot: Information about mechanism pathway
pH dependence	Uniprot: Information about the protein function's pH dependence
Temperature dependence	Uniprot: Information about the protein function's temperature dependence
Cofactor	Uniprot: Information about the protein's cofactors

Supplementary Table 10 continued

Term in database	Description
Kinetics	Uniprot: Information about the kinetics of the protein
mRNA sequence	mRNA sequence of the gene product
AA sequence	Amino acid sequence of the gene product
CIF sequence of # - X	PDB: Amino acid sequence of the chain X in CIF #
Unmapped active residues from CIF #	PDB: Chain ID, Residue # in CIF file, Residue Name, Ligand in contact with
Mapped active residues from CIF #	PDB: Chain ID, Mapped residue #, Residue Name, Ligand in contact with
Ligand abbreviation from CIF #	PDB: 3 letter abbreviation, ligand name
Unmapped interfacial residues from CIF #	PDB: Chain 1, residue name, unmapped residue # in CIF file, Chain 2, residue name, unmapped residue # in CIF file
Mapped interfacial residues from CIF #	PDB: Chain 1, residue name, mapped residue, Chain 2, residue name, unmapped residue # in CIF file
Small molecule active residues (mapped)	PDB: List of residues in contact with small molecules and ligands
Interfacial active residues (mapped)	PDB: List of interfacial residues in the crystal structure
Combined active residues (mapped)	Uniprot and PDB: List of functional residues from Uniprot, residues interacting with small molecules and interfacial residues

Supplementary Table 11. Percent of native state simulation frames with metric ≥10% averaged over set of 122 *E. coli* proteins

Metric	Mean [95% CIs]*, %	Range (low-high), %
$\zeta_{\mathrm{hydrophobic}}(t)$,	6.32 [5.44, 7.27]	0.08 – 32.31
$\zeta_{\mathrm{DnaK}}(t)$	14.74 [13.03, 16.47]	0.17 – 38.35
$\zeta_{\mathrm{agg}}(t)$	11.74 [10.32, 13.22]	0.60 – 39.75
χ_{func}	24.58 [22.97, 26.28]	9.52 – 55.47

*confidence intervals calculated from bootstrapping 10^6 times
