# Supplementary material for: Universal protein misfolding intermediates can bypass the proteostasis network and remain soluble and less functional
Source: Nat Commun. 2022 Jun 2;13:3081. doi: 10.1038/s41467-022-30548-5 (PMC9163053; doi:10.1038/s41467-022-30548-5)
Supplement: Supplementary file 2 — Description of Additional Supplementary Files [file 41467_2022_30548_MOESM2_ESM.pdf]

Description of Additional Supplementary Files

File Name: Supplementary Data 1
Description: Annotated LiP-MS results for GlpD

File Name: Supplementary Data 2
Description: LiP-MS detected peptides for 1-min timepoint

File Name: Supplementary Data 3
Description: LiP-MS detected peptides for 5-min timepoint

File Name: Supplementary Data 4
Description: LiP-MS detected peptides for 120-min timepoint
