# Supplementary material for: Universal protein misfolding intermediates can bypass the proteostasis network and remain soluble and less functional
Source: Nat Commun. 2022 Jun 2;13:3081. doi: 10.1038/s41467-022-30548-5 (PMC9163053; doi:10.1038/s41467-022-30548-5)
Supplement: Supplementary file 7 — Reporting Summary [file 41467_2022_30548_MOESM7_ESM.pdf]

nature portfolio

Corresponding author(s): Edward P. O'Brien

Last updated by author(s): YYYY-MM-DD

# Reporting Summary

Nature Portfolio wishes to improve the reproducibility of the work that we publish. This form provides structure for consistency and transparency in reporting. For further information on Nature Portfolio policies, see our Editorial Policies and the Editorial Policy Checklist.

## Statistics

For all statistical analyses, confirm that the following items are present in the figure legend, table legend, main text, or Methods section.

| n/a | Confirmed | |
|---|---|---|
| ☐ | ☒ | The exact sample size ($n$) for each experimental group/condition, given as a discrete number and unit of measurement |
| ☐ | ☒ | A statement on whether measurements were taken from distinct samples or whether the same sample was measured repeatedly |
| ☐ | ☒ | The statistical test(s) used AND whether they are one- or two-sided<br>*Only common tests should be described solely by name; describe more complex techniques in the Methods section.* |
| ☐ | ☒ | A description of all covariates tested |
| ☐ | ☒ | A description of any assumptions or corrections, such as tests of normality and adjustment for multiple comparisons |
| ☐ | ☒ | A full description of the statistical parameters including central tendency (e.g. means) or other basic estimates (e.g. regression coefficient) AND variation (e.g. standard deviation) or associated estimates of uncertainty (e.g. confidence intervals) |
| ☐ | ☒ | For null hypothesis testing, the test statistic (e.g. $F$, $t$, $r$) with confidence intervals, effect sizes, degrees of freedom and $P$ value noted<br>*Give P values as exact values whenever suitable.* |
| ☒ | ☐ | For Bayesian analysis, information on the choice of priors and Markov chain Monte Carlo settings |
| ☐ | ☒ | For hierarchical and complex designs, identification of the appropriate level for tests and full reporting of outcomes |
| ☐ | ☒ | Estimates of effect sizes (e.g. Cohen's $d$, Pearson's $r$), indicating how they were calculated |

*Our web collection on statistics for biologists contains articles on many of the points above.*

## Software and code

Policy information about availability of computer code

| | |
|---|---|
| Data collection | Experiment: No software was used in data collection. A Thermo Q-Exactive HF-x Orbitrap mass spectrometer was used to analyze protein digests.<br>Computation: simulations were run in CHARMM v35 |
| Data analysis | Experiment: Proteome Discoverer (PD) Software Suite (v2.4, Thermo Fisher) and the Minora Algorithm were used to analyzer mass spectra and perform Label Free Quantification (LFQ) of detected peptides. PD output files were outputted in a three-lebel hierarchy (protein > peptide group > consensus feature) and analyzer with in-house python scripts. These analysis programs are available at https://github.com/FriedLabJHU/Refoldability-Tools/<br>Computation: simulations were analyzed using CHARMM v35, Python, Visual Molecular Dynamics v1.9.1, and the SciPy and PyEmma packages. Custom codes and sample analyses are available at https://github.com/obrien-lab-psu/ |

For manuscripts utilizing custom algorithms or software that are central to the research but not yet described in published literature, software must be made available to editors and reviewers. We strongly encourage code deposition in a community repository (e.g. GitHub). See the Nature Portfolio guidelines for submitting code & software for further information.

## Data

Policy information about availability of data

All manuscripts must include a data availability statement. This statement should provide the following information, where applicable:

- Accession codes, unique identifiers, or web links for publicly available datasets
- A description of any restrictions on data availability
- For clinical datasets or third party data, please ensure that the statement adheres to our policy

The mass spectrometry proteomics data generated in this study have been deposited to the ProteomeXchange Consortium via the PRIDE partner repository with the dataset identifier PXD031425. Summary data for these experiments are also provided in Supplementary Data 1, 2, 3, and 4. We cannot feasibly provide all ~30 TB of molecular dynamics trajectory data, but we do provide sample trajectory files and use them to demonstrate our analysis methods at https://github.com/obrien-lab-psu/. Protein structures were obtained from rcsb.org. Partial functional information was obtained from uniprot.org.

# Field-specific reporting

Please select the one below that is the best fit for your research. If you are not sure, read the appropriate sections before making your selection.

☒ Life sciences ☐ Behavioural & social sciences ☐ Ecological, evolutionary & environmental sciences

For a reference copy of the document with all sections, see nature.com/documents/nr-reporting-summary-flat.pdf

# Life sciences study design

All studies must disclose on these points even when the disclosure is negative.

| | |
|---|---|
| Sample size | Experiment: No sample size calculation was conducted prior. Each experiment was conducted as biological triplicates to allow for statistical analysis.<br>Computation: 6,150 trajectories were run in total, 50 per protein studied. No sample size calculation was conducted, these numbers of trajectories were chosen due to the limitations of our computational resources. |
| Data exclusions | Experiment: No data were excluded from our study and quantifications for all peptides are provided. Sequenced peptides were evaluated for significance based on two criteria, having a greater than two fold difference between native and refolded samples and having a p value < .01.<br>Computation: no trajectories were excluded |
| Replication | Experiment: Limited Proteolysis experiments were conducted on two separate experiments. Experimental findings were consistent in both experiments.<br>Computation: each simulation type was replicated 50 times with different random seeds to allow for the calculation of statistics. Due to different random seeds, behavior is different between trajectories. All trajectories ran to completion. |
| Randomization | Experiment: E. coli lysates were divided into either being either a native or a refolded sample depending on if they were unfolded and refolded or not. Covariates are not relevant in our study as all bacteria are cultured simultaneously under identical growth conditions and simultaneously prepared under identical methods.<br>Computation: molecular dynamics trajectories were run with unique random seeds to generate different trajectories |
| Blinding | Experiment: Investigators know which E. coli lysates are native samples and which ones are refolded samples as limited proteolysis experiments are conducted after different refolding time points. Our analysis compares the limited proteolysis peptide profile of our refolded samples to our native samples, so blinding is not possible.<br>Computation: Blinding not relevant |

# Reporting for specific materials, systems and methods

We require information from authors about some types of materials, experimental systems and methods used in many studies. Here, indicate whether each material, system or method listed is relevant to your study. If you are not sure if a list item applies to your research, read the appropriate section before selecting a response.

## Materials & experimental systems

| n/a | Involved in the study |
| --- | --- |
| ☒ | ☐ Antibodies |
| ☒ | ☐ Eukaryotic cell lines |
| ☒ | ☐ Palaeontology and archaeology |
| ☒ | ☐ Animals and other organisms |
| ☒ | ☐ Human research participants |
| ☒ | ☐ Clinical data |
| ☒ | ☐ Dual use research of concern |

## Methods

| n/a | Involved in the study |
| --- | --- |
| ☒ | ☐ ChIP-seq |
| ☒ | ☐ Flow cytometry |
| ☒ | ☐ MRI-based neuroimaging |
